# Ending the Energy-Poverty Nexus: An Ethical Imperative for Just Transitions

**DOI:** 10.1007/s11948-022-00383-4

**Published:** 2022-08-10

**Authors:** Saurabh Biswas, Angel Echevarria, Nafeesa Irshad, Yiamar Rivera-Matos, Jennifer Richter, Nalini Chhetri, Mary Jane Parmentier, Clark A. Miller

**Affiliations:** 1 Pacific Northwest National Laboratory, Richland, USA; 2 School for the Future of Innovation in Society, Arizona State University, PO Box 875603, Tempe, AZ 85287-5603 USA

**Keywords:** Energy transition, Solar energy, Energy ethics, Sociotechnical systems, Users

## Abstract

Arguments for a just transition are integral to debates about climate change and the drive to create a carbon-neutral economy. There are currently two broad approaches rooted in ethics and justice for framing just energy transitions. The first can be described as internal to the transition and emphasizes the anticipation, assessment, and redressing of harms created by the transition itself and the inclusion in transition governance of groups or communities potentially harmed by its disruptions. In this article, we propose a second approach to ethics and justice in an energy transition, which we describe as systemic or societal in scope. This approach complements attention to the proximate dynamics and impacts of the transition process with a focus on the distant societal and economic outcomes the transition brings into being and how they compare to conditions prior to the transition. It poses the question: do the transformative social, economic, and technological changes wrought by energy systems create more just societies and economies, or do they instead reinforce or recreate long-standing injustices and inequalities? We illustrate this approach with an assessment of one of the most significant existing forms of energy injustice: the energy-poverty nexus. We argue that the energy-poverty nexus reflects configurations of socio-energy systems that create complex, extractive feedbacks between energy insecurity and economic insecurity and, over time, reinforce or exacerbate poverty. We further argue that just energy transitions should work to disentangle these configurations and re-design them so as to create generative rather than extractive feedbacks, thus ending the energy-poverty nexus and creating long-term outcomes that are more just, equitable, and fair.

## Introduction

Arguments for a *just transition* are integral to debates about climate change and the drive to create a carbon-neutral future. Decarbonizing the energy sector involves disruptive changes to both energy technologies and broader societies and economies (Miller et al., 2013, 2015). In the past, energy transitions have wrought major shifts in industries, regulations, workforces, and markets (Jones, 2014), as well as the forms and geographies of resource extraction (Pasqualetti, 2011). As a result, profound ethical questions arise about the meaning and impact of energy transitions for diverse groups of people within and across societies (Jasanoff, 2011, 2018; Miller, 2014; Sovacool et al., 2017). Proponents of a just transition insist, therefore, that the design and navigation of future energy transitions acknowledge and mitigate the resulting disruptions in people’s lives, livelihoods, health, and environments. As the Just Transition Alliance (2020) put it, for example:

“Just Transition” is a principle, a process and a practice. The principle of just transition is that a healthy economy and a clean environment can and should co-exist. The process for achieving this vision should be a fair one that should not cost workers or community residents their health, environment, jobs, or economic assets. Any losses should be fairly compensated. And the practice of just transition means that the people who are most affected by pollution—the frontline workers and the fenceline communities—should be in the leadership of crafting policy solutions.

This definition encompasses both an ethical and a justice-oriented stance. Energy ethics and justice are overlapping fields. Both raise questions about the normative dimensions of energy transitions. Energy ethics is concerned with the social principles, values, and norms that govern energy transitions. Energy justice uses distributive, recognition, and procedural frameworks familiar from environmental justice movements to interrogate and challenge patterns of inequitable distributions of benefits and burdens in an energy transition, focusing especially on historically marginalized populations and communities (Bethem et al., 2020; Bickerstaff, 2017; Biswas et al., 2021; Heffron & McCauley, 2017; Jenkins et al., 2018; McCauley et al., 2019; Jetten et al., 2020; Sovacool & Dworkin, 2015). Questions of distributive justice interrogate how the benefits and burdens of energy systems and transitions are distributed across groups. Recognition justice “refers to the acknowledgement of, and respect for, the complex circumstances and vulnerabilities of individuals and social groups in patterns of cultural value” (Lewis et al., 2020, p 425–426). Procedural and participatory justice focus on how decisions get made, the differential abilities of and opportunities for groups to contribute to and shape decision-making, and the institutional barriers that limit or constrain their participation.

Two broad approaches rooted in ethics and justice currently frame discussions of just energy transitions, and are present in the Just Transition Alliance quote above. The first can be described as *internal to the transition* and emphasizes the anticipation, assessment, and redressing of harms created by the transition itself and the inclusion in transition governance of groups or communities potentially harmed by its disruptions. This approach tends to focus on current coal, oil, or gas workers whose employment is threatened (Pai et al., 2020; Pai & Carr-Wilson, 2018, 2019); communities that may lose tax revenues or businesses due to the decline of these industries or confront environmental risks from legacy infrastructures stranded by the transition (Roemer & Haggerty, 2021); or communities confronting challenges due to the siting of new energy infrastructures, such as lithium mines, solar and wind farms, or natural gas wells (Barandiaran, 2015; Kinchy, 2017; Mulvaney, 2019).

In this paper, we focus on a second approach to ethics and justice in energy transitions, which is *systemic or societal* in scope. This approach complements attention to the *proximate* dynamics and impacts of the transition itself with a focus on the *distant* societal and economic outcomes the transition brings into being and how they compare to conditions prior to the transition. It poses the question: *do the transformative social, economic, and technological changes wrought by energy transitions create more just societies and economies, or do they instead reinforce or recreate long-standing injustices and inequalities?*

The present reality is that carbon-based energy systems systematically help create and foster a wide range of structural inequalities and injustices across markets and societies (Sovacool, 2016). We propose, therefore, that those leading energy transitions have an ethical obligation to redress these existing inequalities and injustices, in addition to proactively addressing any potential additional harms created by the transition process, thus contributing to more just, equitable, and fair societies and markets. This can be accomplished by using ethical and justice principles and practices to inform the (re)design of energy systems and the work of carrying out energy transitions by institutions in the energy sector and with regulatory or governance authority over energy, as well as by civil society organizations and communities working with them. Ultimately, we argue, just transitions to carbon-neutral energy systems should promote the creation of alternative socio-energy arrangements that intentionally create and improve positive social, political, economic and environmental conditions and outcomes for the world’s lowest income and least well-off communities, rather than continue to diminish them.

We hope that the issues raised in this paper will resonate with policy-makers, planners, and designers at local, state, regional, and international levels, including energy professionals as well as community leaders and members. Our ideas are relevant both to those engaged in efforts to plan for and implement carbon-neutral energy, as well as those seeking to localize energy design to address other structural issues, such as food insecurity or climate adaptation (Lewis et al., 2020). Our analysis should also resonate with researchers seeking to integrate equity and justice into energy modeling (Mayfield et al., 2019) or working with grassroots organizations and communities to help those most affected by energy transitions (Rivera Matos et al., 2021).

## Defining the Energy-Poverty Nexus

To illustrate our approach to just transitions, we examine in this paper what we consider to be one of the most important existing facets of the problem of energy injustice and inequality: *the energy-poverty nexus* (Biswas, 2020; Biswas et al., 2020). Consistent with our overall argument above, we suggest in the rest of this article that the energy transition offers a unique opportunity to reduce the contribution of energy systems to enduring poverty and inequality and, ideally, to leverage new energy system designs to foster improved economic security and wellbeing in low-income households and communities.

We define the energy-poverty nexus as an ensemble of complex, negative feedbacks in socio-energy systems (Miller et al., 2015) that, over time, reinforce and exacerbate poverty and make conditions worse off for individuals, households, or communities. Our definition of the energy-poverty nexus incorporates but extends beyond the idea of energy poverty. Energy poverty typically refers to a lack of access to the energy or fuel resources necessary to create a good life (González-Eguino, 2015; Jenkins et al., 2016). This lack of access may arise from a variety of causes (Sovacool, 2012). In developing countries, for example, it may stem from the absence of technological access to modern energy services, such as reliable and sufficient electricity or clean cooking technology, although affordability is also often a problem (Pauchari et al., 2004). In wealthy countries, it generally results from the lack of ability to pay for energy or a high energy burden in which households pay a large proportion of income for energy (Bouzarovski et al., 2012; Lewis et al., 2020).

In both developing and wealthy countries, considerable research has begun to illuminate that the concept of energy poverty, as traditionally defined, underestimates the complexity of the problems facing communities vis-à-vis energy (Lewis et al, 2020). Many marginalized communities not only face difficulties accessing and affording energy, but they also pay higher costs and higher fractions of their income for energy and confront a suite of complex and intertwined forms of energy insecurity. Elements of this insecurity include: poor quality energy equipment and infrastructures, with little financial capacity to improve them; more substantial and longer duration disruptions of energy systems after disasters, e.g., as occurred for rural and remote communities in Puerto Rico after Hurricanes Irma and Maria (Castro-Sitiriche, 2018); debilitating trade-offs between energy and food and nutrition, health, education, and employment (Caniglia et al., 2016); disproportionate environmental and health burdens and risks from energy systems; and unjust governance processes that more frequently site pollution-generating facilities in or nearby to low-income communities, communities of color, and indigenous communities (Bullard, 2008; Sze, 2006). Thus, the organization of socio-energy systems reinforces and reinscribes poverty and inequality in communities that lack the economic or political resources or power to change how these systems work. Social and economic insecurities thus perpetuate and exacerbate energy insecurities that, in turn, further undermine social and economic wellbeing and capabilities, creating a cycle of value destruction, structural injustice, and unequal opportunities (Liu et al., 2015; Samad et al., 2010).

The value of adopting *a socio-energy systems approach* to analyzing these dynamics is its ability to capture these reinforcing feedback loops and interwoven, interdependent interactions among energy systems, social and economic insecurities, and environmental degradation within and across communities. Over the past several decades, researchers have observed that electricity, transportation, and fuel systems interweave social, economic, and technological elements (Geels, 2002; Hughes, 1983; Jasanoff & Kim, 2013; Jones, 2014; Nye, 1996; Winner, 1986). The resulting socio-energy systems operate across multiple scales, from daily social practices at the level of individuals and households (Shove et al., 2012) to national imaginaries and identities (Jasanoff & Kim, 2015; Hecht, 1998) to planetary geopolitics and international security (Sovacool & Dworkin, 2014). Crucially, these relationships are bi-directional: social structures, values, and dynamics both shape and are shaped by the design and operation of energy systems (Allison, 2015; Mitchell, 2011; Ottinger, 2013a, 2013b). It is this bi-directional reinforcement of insecurities that makes understanding and solving the energy-poverty nexus so difficult.

It is also important to take *a multi-faceted approach to poverty*. Lack of income or assets is often used to measure poverty, including as a proxy for the wider array of opportunities available to individuals, households, and communities in market-based societies (Perry, 2002; Triest, 1998). Even so, many aspects of poverty cannot be reduced to money (Hick, 2014). As an alternative, Sen and others have focused on the capabilities and freedoms of individuals, households, and communities to imagine, shape and pursue development objectives and control their own futures (Nussbaum, 2011; Sen, 2001). The *capabilities approach* refines the understanding of poverty in two critical ways: first, the knowledge, skills, and capacity to act can never be reduced entirely to income; and, second, individuals, families, and communities are not necessarily free to exercise their capabilities but instead often face externally imposed structural conditions that limit the ability to plan and execute improvements in their lives and livelihoods (Middlemiss et al., 2019). In our experience working and conducting research in collaboration with communities around the globe, both aspects of poverty contribute to the energy-poverty nexus, often in mutually reinforcing ways.

## Untangling the Energy-Poverty Nexus

Pulling these ideas together, we approach the energy-poverty nexus in terms of configurations of socio-energy systems that deepen, reinforce, and exacerbate a variety of factors that either reduce the income of communities, undermine their capabilities to improve their wellbeing and livelihoods, or both (Drehobl & Ross, 2016; Gómez-Barris, 2017; Moore, 2013). We term such configurations *extractive*. In pursuing a just transition, we believe that societies have an ethical obligation to redress the forms of injustice reflected in the energy-poverty nexus. At a minimum, the obligation is to design future socio-energy systems that no longer exacerbate poverty. Ideally, the obligation would be to design socio-energy systems that are *generative*: i.e., that support low-income communities over time in their quest to escape poverty and achieve higher quality of life. The latter is what we mean by the phrase *to end the energy-poverty nexus*.

Many facets of socio-energy system design contribute to the energy-poverty nexus. Here we describe three major categories of design elements that work together to create and exacerbate negative feedbacks between energy insecurity and economic insecurity. In the first, *equity among energy users*, we examine the commitment of socio-energy systems to the principle of equity and the ways in which that commitment too often falls short, reinforcing inequalities and thus undermining the ability of low-income communities to use energy to alleviate or escape poverty. In the second, *positionality within energy supply chains*, we highlight the structural organization and design of energy systems and the ways in which that structural design distributes risks, harms, benefits, and opportunities within energy systems. Finally, in the third category, *capacity in energy systems governance*, we focus on the political design of energy systems and the ways in which governance institutions distribute capacity, empowerment, and self-determination.

To illustrate these three dimensions of the energy-poverty nexus and how a focus on socio-energy system design reveals them, we synthesize insights from research streams that we have been involved in over the past decade. These include: (a) projects to develop community-based energy systems and innovation in Puerto Rico in the aftermath of Hurricane Maria, which destroyed Puerto Rico’s electricity grid in 2017; (b) rural electrification projects in Asia and Africa, where we have worked with communities and small renewable energy project developers to leverage energy development to tackle poverty and inequality; and (c) research on the experiences of indigenous communities in the US Southwest who are grappling with the challenges of inhabiting and working within landscapes shaped by resource extraction from uranium, coal, oil, and gas and energy other aspects of energy resource development, as well as nascent renewable energies.

### Equity Among Energy Users

The principle of equity has been enshrined in energy systems design predominantly through two broad commitments: that all users should have energy available to them to use, usually referred to as *the principle of access*; and that all users should pay similar, low rates for that energy, and especially for basic energy services, usually referred to as *the principle of affordability* (Fankhauser & Tepic, 2007). These principles provide the core justification for treating energy as a basic right, and they inform the UN SDG7 goals of providing universal access to affordable, clean energy. Principles of access and affordability have been central to shaping both electricity and fuel markets (Walker & Day, 2012). Electric utilities are generally required, for example, to provide electricity to everyone at the same price. In many countries, the principle of equity has also underwritten the expansion of universal electricity service to rural communities.

Over time, however, it has become increasingly clear that this approach to equity in energy system design is insufficient to create equitable outcomes and, in some contexts, creates or exacerbates conditions of inequity and injustice (Heindl & Schuessler, 2015). The problem, in our view, is that conventional approaches to equity adopt *a supply-side perspective*: they concern equity in the availability and price of energy supply. By contrast, we suggest, *a user-centered perspective* can help make more visible a range of problematic aspects of socio-energy systems, especially in terms of unequal or inequitable *outcomes* for different groups of users and the ability of users to participate in and contribute to decisions that impact those outcomes. Energy users care about affordability and access, but they also care about their needs for energy and what they are able to do with the energy they have access to, the risks and benefits of energy use, and how decisions about energy systems get made and impact them.

The impact of energy costs on different users, for example, depends not only on the price of energy but how that price fits into the users’ financial circumstances. Extreme energy burdens, defined as the fraction of a household’s or community’s monthly income that goes to pay for energy, are arguably one of the most inequitable elements of existing socio-energy systems. Across a wide range of communities, high energy burdens drain financial resources from households, severely impacting wellbeing and opportunity. Such burdens often create trade-offs between paying energy bills and securing necessities like food or healthcare, while also limiting available funds for investing in education, businesses, or other pathways to improved livelihoods. In general, the need for basic infrastructure services like energy, housing, water, and food isn’t proportional to income, and hence low-income communities typically commit more of their resources to securing them. This is especially true for rural, remote, and other low-income communities (Bazilian et al., 2014).

In Puerto Rico, for example, US Census data shows that tens of thousands of households with less than 30% of area median income (many of whom lie in the remote, mountain interior) confront combined electricity and natural gas costs as high as 40–50% of their monthly income. Adding in the cost of gasoline (Puerto Rico has poor public transport, leaving almost everyone dependent on automobiles) and the energy costs embedded in food and other purchased goods (most of which are imported to Puerto Rico) further increases household energy burdens to extreme levels. Other low-income communities in the US and Europe face similar challenges. Electricity and natural gas burdens for the lowest 20% of households by income in major US cities are 2–4 times higher than for the population as a whole. For the lowest 10% of households by income, across the US as a whole, the total energy burden (not including embedded energy costs) is 35%. Indigenous communities across the US and Canada (especially in northern regions, where they experience extremely cold winters) rely for both power and heating on costly diesel fuel (in remote areas of the Arctic, for example, these fuels are flown in at extremely high expense) (Karanasios & Parker, 2018). In developing countries, e.g., in South Asia, electricity from the grid often remains too costly for low-income communities. Consequently, they often eschew grid electricity in favor of local alternatives, such as small hydro or solar home systems. Or, they continue to rely on kerosene or other fuels, which are expensive and contribute to high energy burdens, but whose cost can more easily be controlled as they are point of sale rather than after-the-fact monthly bills (Balachandra, 2011; Islar et al., 2017; Williams et al., 2017).

High energy burdens in South Asia and elsewhere are further compounded in many communities by low quality energy infrastructures. For nearly half of the world’s electrified populations, grid infrastructures are in poor shape. Utility debt, lack of public or private investment, and associated poor maintenance and upkeep all increase the unreliability and low quality of electricity services, resulting in frequent disruptive outages, poor voltage and frequency control, damage to household equipment, and other challenges. These disruptions reduce access to energy while often forcing low-income households to purchase additional equipment, such as voltage control devices to protect equipment or backup generators to compensate for outages, as well as the diesel or kerosene to run them, and also to frequently pay high costs to repair or replace damaged end use devices (see, e.g., Aklin et al., 2020; Ahmad et al., 2016; Khokhar et al., 2015). Limited ability to invest in new electrical equipment and repairs also often forces households to use low-efficiency appliances, resulting in higher overall energy consumption per task. At the same time, low-quality electrical junction boxes or poorly maintained roofs prevent households from acquiring more efficient devices, solar systems, or other technology upgrades that might reduce future electricity bills (see, for example, Patnaik & Jha, 2020; Hernandez, 2013; Reames et al., 2018).

Unfortunately, policies to address high energy burdens often fail to reach the very lowest income communities, especially when grounded in subsidies or incentives that presuppose substantial financial investment on the part of the energy user. A common practice in the energy sector, for example, is to offer subsidies for the purchase of more energy efficient equipment (e.g., high-efficiency water heaters) or new energy technologies (e.g., rooftop solar systems or electric vehicles). For low-income communities, however, these technologies typically remain too expensive, even with substantial subsidies, due to up-front capital requirements. Thus, the benefits of subsidies generally flow to middle and higher income households. Similarly, subsidized electricity use in developing countries, which is often allocated per unit electricity consumed, distributes the least benefit to the lowest income households, who traditionally use significantly less electricity. Program designs that prioritize the lowest income groups are possible but rare. For example, subsidies can target the first tranche of electricity use, or governments can directly purchase and install high-efficiency equipment for low-income households (Cardenas & Whittington, 2019; Mayer et al., 2015; He & Reiner, 2014).

Inequity also arises in the capabilities of energy users to create value for themselves and their communities through the use of energy (Miller et al., 2015, 2018). Only if all users are able to use energy to generate high social or economic value will outcomes be equitable. Unfortunately, many policies in developing countries focus too narrowly on increasing household use of electricity. This often creates incentives for households to use electricity in non-productive ways, e.g., by adding unnecessary lighting or, more egregiously, giving away televisions or offering tailored loan products to new customers to encourage high energy consumption, setting off a cycle of dependency (Rolffs et al., 2015). Alternative approaches put greater emphasis on enhancing users’ capabilities. This requires growing energy services that improve social and economic value creation, such as creating new business opportunities. It also requires ensuring that users pay equitably for those energy services (e.g., by having equitable access to high efficiency equipment) and have the knowledge, skills, tools, and opportunities to capture those opportunities. At higher levels of organization, redesigned energy systems can also create new opportunities for communities, e.g., via local energy workforce development, new approaches to energy ownership, and policy reform (Miller et al., 2018).

To return to our primary argument: shifting from a supply-centered to a user-centered perspective illuminates that simply equalizing energy supply and cost can still result in some households and communities becoming poorer and more economically insecure over time. This, in our view, is ethically problematic. Energy systems, from a user-centered perspective, should be designed so that they are economically generative and not economically extractive.

### Positionality Within Energy Supply Chains, Infrastructures, and Waste Streams

The second major dimension of the energy-poverty nexus arises from the positionality and agency of individuals and groups within the geographies of socio-energy systems. Energy systems have extensive infrastructures, supply chains, and lifecycles that structure people’s positions within them and the benefits, costs, risks, and burdens they experience. Oil is mined, transported, refined, piped, and then burned, for example. Materials to make solar panels are mined, transported, and used to manufacture panels, which are then transported, installed, and, at the end of their useful life, discarded or recycled. Electricity is generated at power plants, transmitted and distributed via grids, and used in homes and businesses. People are situated throughout these systems in ways that, in many cases, expose them to a variety of economic, social, or environmental risks and insecurities but leave them little opportunity to influence or control system-level decisions. The fact that the world’s economies and societies depend heavily on the smooth functioning of these systems tends to further reduce people’s capacity to alter their design and operation in more favorable ways.

Principles of recognition justice emphasize the dearth of attention given to historically vulnerable and marginalized groups who inhabit energy systems and geographies and the systemic issues that contribute to the forms of energy-poverty nexus they experience (Hernandez et al., 2022; Lewis et al., 2020). The problem isn’t just recognition of these challenges, however, but the redesign of systems to ensure they are generative throughout. Like financial systems, energy systems are often deemed “too big to fail” and seen as critical infrastructures that underpin national security, the health of the economy, and human wellbeing. The imperatives of ensuring high reliability and expanding energy systems to provide energy to growing economies often overshadow other concerns, leading to a variety of problematic relationships within socio-energy systems that leave disadvantaged groups to bear the brunt of energy production, transport, and consumption and often with little opportunity to voice their concerns.

One of the most visible and obvious dimensions of this positionality relates to ownership and control over the resources for producing energy. Over the past two centuries, as energy systems have come to rely on extracted materials (primarily carbon-based fuels like coal, oil, and natural gas, but also uranium for nuclear power plants), wealth in the energy industry has become highly concentrated among those able to assert control over those resources, epitomized by the oil kingdoms of the Middle East, billionaire coal and oil magnates across the Americas and Asia, and the concentration of capital in a handful of oil and automobile companies that were, for much of the twentieth century, the largest companies in the world (Mitchell 2009). By contrast, many developing countries and regions have suffered for decades from high energy dependence and debt created by the extractive economic relationships embedded in energy supply chains. For example, $3 billion leaves Puerto Rico each year (5% of annual economic activity) to pay for carbon to burn in cars and power plants, exacerbating the territory’s high debt levels and colonial relationships with the United States (EIA, 2020).

The imperatives of energy development and security have also driven the creation of widespread *sacrifice zones* throughout energy supply chains, impacting a variety of different communities and leading to the well-known phenomena of *the resource curse*: energy development is meant to provide an economic boon to local development, yet many energy communities suffer from systemic poverty and precarity that leaves them worse off over time, economically, environmentally, and politically (Adams et al., 2019; Frankel, 2010). Workers in many energy communities are exposed to deadly health risks (e.g., in coal and uranium mining). Energy development has contributed to widespread environmental destruction, pollution of air and water, and political conflict and violence. Resource-based local and state economies are frequently at the mercy of both energy resource owners and changes in far-flung energy markets and policies that, as in coal communities today, can create massive economic disruption (Haggerty, 2017; Ladd, 2018). In our view, energy systems that rely on precarity and scarcity for their growth are both unsustainable and unjust.

As an example, indigenous communities such as the Navajo Nation confront a confluence of risks (Curley, 2018; Pasqualetti et al., 2016). The Four Corners area of the American Southwest is rich in resources, including uranium and coal, and both have left an indelible mark on the land and people of the region. In the 1950s, the discovery of uranium deposits on Navajo land led to an intense period of speculation and mining by uranium companies, who were backed by guaranteed federal buyers and prices. Navajo miners were told that they would be able to stay close to home while earning much-needed paychecks. They were not warned, however, of the risks from radiation in the uranium dust they were inhaling and bringing home to their families (Brugge et al., 2006; Voyles, 2015). When the uranium boom collapsed in the late 1960s, companies went bankrupt and left the region, abandoning hundreds of mines, many of which still have not been addressed (EPA, 2018). Cancers and other health concerns began to spike among the Navajo, who had to rely on the underfunded and remote Indian Health Services for information and health care (Brugge et al., 2006). The legacy of radioactive contamination across the Navajo Nation still reverberates, and the Navajo banned uranium mining on all tribal lands in 2005 (Powell, 2018). They still grapple, however, with persistent health and environmental concerns, as well a persistent lack of trust in governmental agencies (Dawson & Madsen, 2007).

These concerns were compounded by the development of coal in the 1970s on Black Mesa. Northeastern Arizona hosted five coal power plants, including the Navajo Generating Station in Page, Arizona, which closed in 2019. The plants were supplied by the Kayenta Mine, located on indigenous land, but mine owners negotiated extremely poor long-term contracts which the Department of the Interior approved, exploiting a lack of understanding and context on the part of Navajo and Hopi communities (Powell, 2015). This pattern of development has systematically devalued indigenous resources, while profiting corporations that extract resources (Allison, 2015; Wilkins, 2013). Similar patterns have occurred elsewhere. When strip-mining became more economical than underground mining in Appalachia, for example, coal communities were abandoned for more easily-mined and lower-sulfur coal in the US West, including Wyoming and the Four Corners area (Jones, 2014). Currently, coal is less profitable than natural gas, and is also recognized as a key contributor to climate change, leading to a rash of plant and mine closures across the US. The sudden closures of facilities such as mines or plants that communities rely on for economic stability is a wrenching change, leaving them with few economic options, even as they must now wrestle with the legacies of contamination, pollution, and abandoned infrastructures (Roemer & Haggerty, 2021).

To end the energy-poverty nexus, therefore, requires attention throughout socio-energy systems to where the costs, risks, and burdens borne by different groups of people outweigh the benefits—recognizing that this calculus is different in different positions within the system—and working to redesign systems so that they are generative across all positions.

### Capacity in Energy Systems Governance

The third major contributing element to the energy-poverty nexus is *a lack of capacity to participate meaningfully in the governance of energy systems*. Communities often lack the authority, opportunity, and ability to influence energy decisions as well as the knowledge and skills to do so effectively, both of which undermine their capacity to exercise self-determination with respect to imagining, designing, implementing, or operating energy systems. This lack of capacity creates and exploits participatory and procedural injustices, limits access to reliable and timely information, reduces transparency in the decision-making process, and directs attention away from efforts to redress the harmful legacies of energy production (Hernandez et al., 2021). Higher-level systems analyses often ignore or erase the distinctive contexts and organization of local energy communities and the “moral economies” (Curley, 2019) and cultural cosmologies (Smith Rolston, 2014) that shape their willingness and ability to engage in energy development and to participate in market and policy decisions. To address the energy-poverty nexus, careful attention is needed, therefore, to acknowledge and incorporate community values around labor, land, and energy; to enhance the governance capabilities of marginalized communities; and to expand opportunities for them to exercise those capabilities in energy governance.

In Puerto Rico, for example, energy governance retains significant legacies from the period of colonial rule, with decision-making exercised in non-transparent processes within the executive branch of the territorial government, influenced heavily by external stakeholders in the US financial industry (which controls Puerto Rico’s public sector debt, including the heavy debts of the electric utility), the energy industry (which controls the sources of oil, coal, and natural gas, as well as independent power plants, on which Puerto Rico depends for energy), and the federal government (de Onis, 2018). In recent years, the lack of transparency and self-determination has been exacerbated by the Puerto Rico Oversight Management and Economic Stability Act (PROMESA), through which the US Congress exerted control over Puerto Rico’s finances and budget and subjected them to the decisions of an external fiscal oversight board that is unaccountable to Puerto Ricans, who are not represented by a member of Congress. To help pay the territory’s debt, the fiscal oversight board authorized the privatization of parts of the electric utility’s assets and services, as well as the conversion of power plants from oil to natural gas (Smith-Nonini, 2020). This decision perpetuates dependence on imported carbon-based fuels for additional decades and contradicts legislation recently passed by the Puerto Rican legislature that requires Puerto Rico to achieve 100% renewable energy by mid-century.

In response to these trends, in some rural and remote areas of Puerto Rico, and in communities located near fossil fuel generating stations in the south of the archipelago, a number of communities have been inspired by the potential of solar energy to create alternative power systems (Rivera-Matos et al., 2021). This new imaginary was sparked by the aftermath of Hurricanes María and Irma, which decimated the Puerto Rico electricity grid in 2017 and left hundreds of thousands of people without power for as long as eleven months. Communities now see solar as the foundation for socio-energy systems that are more resilient, foster local energy development, empower local sovereignty over energy decisions, and retain energy finances within the local economy. This ideal is reflected in Queremos Sol (2021), literally “we want the sun,” a collective composed of local experts, environmental activists, lawyers, and people from across Puerto Rico who propose a transition to alternative energy that decentralizes both energy technologies and governance through the development of community solar projects and microgrids.

As in many other parts of the world, community solar in Puerto Rico is driven by a vision of bottom-up energy innovation in low-income communities for whom rooftop solar systems for individual households remain largely out of reach financially. One prominent example is El Coqui and the Iniciativa de Eco Desarrollo de Bahía de Jobos, who have mobilized community-based solar energy projects in communities who have faced decades of environmental and health risks from the nearby coal-fired power plant (Estrada-Garcia, 2016). Casa Pueblo is another organization that has long opposed projects to develop mining and gas that threatened ecosystems in the central mountain region. Recently, they have been working to build “a solar energy revolution” in the community of Adjuntas that leverages rooftop energy to support local businesses and low-income households. Veguita Zama is a community located in Jayuya, another small town within the central mountain range, which has been organizing an alternative solar energy project in collaboration with the Alliance for Sustainable Resources Management, known locally as AMANESER 2025. The initiative focuses on dignity and power for the people living in the “ruralia” through a bottom-up transition approach, using solar and batteries as emergency systems for backup power. They purchase solar as a group, learn to install the equipment, and, importantly for the community, avoid accumulating debt and reliance on external investment or financing.

Given their experiences with destruction wrought by the aftermath of the 2017 hurricanes (and more recently with earthquakes in the region, too, which also disrupted electrical power systems and the vital water, communications, and other infrastructures that depend on them), these communities see “the struggle to transform Puerto Rico’s flawed energy grid with locally-controlled alternatives [as] a matter of life and death” (Santiago et al., 2020). Communities are learning from the injustices embedded in the design of existing socio-energy systems that they need to transform their energy system to thrive socially and economically. They are acting locally and setting examples of how to use new socio-energy arrangements to catalyze social, economic, and political change and end oppression. However, also like many other similarly marginalized communities around the globe, they are struggling to develop the capacity to pursue independent energy development, including: 1) controlling planning and development of resources; 2) acquiring information from trusted and independent sources on both the benefits and risks of diverse energy systems; 3) developing local expertise (including construction, planning, management); 4) addressing immediate community needs while sustaining long-term planning for energy development and access; 5) identifying and securing the financial resources necessary for alternative energy development, and, 6) confronting barriers to local energy innovation put in place by the energy sector, government regulators, and others who are enrolled in the design and operation of centralized energy systems.

If the goal is ultimately to end the energy-poverty nexus, then these limits to capacity are problematic, ethically, not merely because they are unequal (which they are) but because they limit what communities can do to end the energy-poverty nexus.

## Discussion: Creating Generativity to Disrupt the Energy-Poverty Nexus

The current transformation of energy systems from carbon-based to carbon-neutral fuels and technologies offers a once-in-a-century opportunity to not simply redesign of socio-energy systems to address climate change but to also redesign them in ways that reconfigure the relationships between energy and poverty.

At stake in clean energy transitions are the vast majority of the world’s energy resources, the supply chains and systems that transform those resources into usable energy, the industries and organizations that control energy resources and operate energy systems, and the use of that energy to power societies and economies worldwide. Worldwide, electric utilities and the oil, coal, and gas industries all face existential risks as several transitions unfold, and many parts of the global financial sector are confronting the consequences of massive intertwining of monetary and energy systems.

Aspects of these systems and industries may well survive. However, as is illustrated by the above example of Puerto Rico’s bottom-up energy initiatives, renewable energy resources, technologies, and imaginaries also contain at least the potential to nucleate vastly different designs, organization, and governance of socio-energy systems. Solar energy, in particular, can be configured into radically different socio-technological forms, including, e.g., solar-powered devices, like lanterns, lights, and libraries (Linzy & Hosman, 2018); rooftop and solar home systems; community-scale solar; and utility-scale power plants. As such, it offers an especially fertile ground for rethinking the human relationship to energy (Miller et al., 2018).

What would it take to leverage this opportunity to redesign socio-energy systems so that they are generative of more equitable conditions in terms of environmental sustainability, long-term economic thriving, and community health and self-determination, in order to interrupt and end the energy-poverty nexus? In our view, it will take new strategies for identifying, analyzing, and reversing the negative feedback loops that link energy insecurity with economic, social, human, and political insecurity and, instead, leveraging the design of energy systems to grow wellbeing and thriving for communities. Key to this are three innovations, each of which derives from the ideas discussed in this article.

The first, which directly addresses the challenges of equity among the inhabitants of socio-energy systems, including but not limited to energy users, is to leverage a user-centered perspective on energy to ensure that *the ability of people and communities to create value through engagements with energy systems is equitably distributed across all communities and, for all, outweighs the costs, burdens, and risks that energy systems impose*. This is our definition of generative. The system is generative of benefits that outweigh costs, burdens, and risks. For all communities, in other words, energy systems should be generative of improved conditions over time, not extractive and destructive to community cohesion: net benefits should flow in rather than just out.

The second, which addresses positionality amidst energy infrastructures, is *to disperse energy ownership more equitably and much more broadly*. Major aspects of the energy-poverty nexus flow from the concentration of ownership over resources, assets, systems, and opportunities and the resulting concentration of wealth and power. Key to this will be the future of ownership over the billions of solar panels required to power the global economy in a post-carbon future and the sunlight they capture. Solar panels are literally the next oil. But energy ownership will also be at stake in a wide array of other facets of the future of energy and transportation, as it has been historically in debates over, e.g., public versus private power and state versus private ownership of oil.

Finally, the third innovation is *to grow local capacity to engage in and ideally control energy governance*. Understanding the full ramifications of energy development, with regard to both existing centralized, carbon-based energy systems and future alternatives, “requires ethnographic attention to the moral commitments, people, and practices that constitute and defend it” (Smith, 2014, p. 36), an exercise that is made significantly easier when people positioned within energy systems have greater power to effectively govern their own energy affairs. This may be the most difficult innovation of all, as it requires finding ways to balance local decision-making with the demands for high-levels energy security and/or to decentralize socio-energy systems to reduce the costs of systems failures (although decentralization, too, comes with its own challenges in terms of achieving universal energy rights and responsibilities, as existing electricity systems illustrate).

There is a pressing and immediate need to rethink what our energy systems should be generating beyond accessibility and affordability. Our research demonstrates that the energy-poverty nexus poses deep challenges at the intersection of energy systems and community life. Only by unpacking how the energy-poverty nexus works in each community and designing alternative arrangements that are more generative of local value creation, ownership, and control can the energy-poverty nexus be ended. The specific dynamics of the energy-poverty nexus vary quite significantly from place to place, even as the general analysis holds that energy insecurities and economic insecurities tend to reinforce one another in strong feedback dynamics. Thus, while solutions must be tailored to the particular situations of any given community, the broad framework holds of redesigning energy systems so that they work to enhance human capabilities and empower value creation that outweighs their costs and risks.

## Conclusion

Energy production and access are often posited as a solution to poverty. Yet many of the consequences of energy system design and operation play a persistent role of in exacerbating and perpetuating poverty, both in a financial sense and, more broadly, in terms of human capabilities. As societies seek to transform energy systems to mitigate climate change, now is the time to focus on how to ensure that the energy systems of the latter half of the twenty-first century and beyond do not continue to replicate those same inequalities and injustices. A major accomplishment of a just transition to a new global energy future should be to design and implement carbon-neutral energy systems that also act to help the worst-off communities determine their own energy futures, alleviate poverty, and grow capabilities by disentangling and ending the energy-poverty nexus.
